# No transport? No worries! Cardiac telerehabilitation is a feasible and effective alternative to centre-based programs

**DOI:** 10.1007/s10741-023-10301-w

**Published:** 2023-02-18

**Authors:** Rita Hwang, Elise M. Gane, Norman R. Morris

**Affiliations:** 1 Department of Physiotherapy, Princess Alexandra Hospital, Metro South Health, Ipswich Road, Woolloongabba, QLD 4102 Brisbane, Australia; 2 School of Health and Rehabilitation Sciences, The University of Queensland, Brisbane, Australia; 3 School of Allied Health Sciences and Menzies Health Institute, Griffith University, Gold Coast, Australia; 4 Centre for Functioning and Health Research, Metro South Health, Brisbane, Australia; 5 Allied Health Research Collaborative, The Prince Charles Hospital, Metro North Health, Brisbane, Australia

**Keywords:** Cardiac rehabilitation, Heart failure, Telemedicine, Home-based, Exercise

## Abstract

Given the under-utilisation of cardiac rehabilitation despite its benefits, there has been a shift towards alternative delivery models. The recent coronavirus disease 2019 (COVID-19) pandemic has accelerated this shift, leading to a growing interest in home-based cardiac rehabilitation including telerehabilitation. There is increasing evidence to support cardiac telerehabilitation, with studies generally demonstrating comparable outcomes and potential cost-benefits. This review aims to provide a synopsis of the current evidence on home-based cardiac rehabilitation with a focus on telerehabilitation and practical considerations.

## Background

Exercise-based cardiac rehabilitation programs should be considered an essential part of the contemporary management of heart failure (HF). Several systematic reviews have confirmed the benefits of these programs, including improved quality of life, reduced hospitalisations in people with HF [1], and their cost-effectiveness [2]. Traditionally, exercise programs for HF have concentrated on centre-based rehabilitation programs, which are usually delivered in hospitals, rehabilitation centres and community facilities [3]. For example, in a pre-pandemic survey of 170 European cardiac centres with 77,214 individuals with HF, exercise-based rehabilitation programs were predominately offered as outpatient (52%) and/or inpatient (25%) centre-based programs, whereas home-based programs were only offered in 18% of the facilities [4]. Whilst these centre-based programs are effective, program uptake and attendance remain challenging. For instance, only 24% of eligible individuals participated in cardiac rehabilitation in the USA [5] and 12% of individuals with HF were referred to cardiac rehabilitation in the UK [6]. In a systematic review of 34 qualitative studies of 1213 individuals, reported barriers to accessing these cardiac rehabilitation programs include a lack of transport and parking, financial cost and competing work and care for others commitments [7]. Alternative delivery models such as home-based cardiac rehabilitation programs may supplement traditional programs and help to overcome some of these geographical and transport barriers. Furthermore, even using the most optimistic modelling of significant expansion of all existing cardiac rehabilitation programs, there is still an insufficient capacity to meet the needs in the USA [8]. Cardiac rehabilitation programs will need to expand beyond the confines of centre-based cardiac rehabilitation, to reach the goal of a 70% participation rate as proposed in the US Million Hearts Initiative [9] and an 85% participation rate as in the UK’s NHS Long-Term Plan [10]. Solutions will likely require the creation of new cardiac rehabilitation programs, improved funding, and expansion of alternative delivery models of cardiac rehabilitation [8]. In recent years, there has been a shift towards alternative delivery models including home-based cardiac rehabilitation programs. The coronavirus disease 2019 (COVID-19) pandemic has accelerated this shift, leading to an increasing interest in telerehabilitation. In this review, we provide a synopsis of the current evidence on home-based cardiac rehabilitation with a focus on telerehabilitation and directions for future research. This review draws on the HF literature where possible, but also relies on the broader cardiac rehabilitation literature for cardiovascular disease.

## Home-based cardiac rehabilitation

In light of the suboptimal uptake of cardiac rehabilitation despite its benefits, there is a growing interest in home-based cardiac rehabilitation. In a Cochrane systematic review of 23 trials with a total of 2890 individuals, home-based cardiac rehabilitation programs demonstrate comparable effects on mortality, exercise capacity, modifiable risk factors and health-related quality of life compared with centre-based programs in people who have suffered a myocardial infarction, angina, HF or who have undergone revascularisation [11]. Hospitalisations and costs have also been shown to be similar between home-based and centre-based cardiac rehabilitation programs, with higher completion rates in the home-based group [12]. Given this lack of difference in clinical outcomes between models, international guidelines have recommended aligning the choice of centre-based or home-based cardiac rehabilitation services with an individual’s needs and preferences [13–15]. However, home-based rehabilitation programs are often delivered one-on-one with a clinician or completed without any supervision or the group interactions associated with centre-based programs. Some authors have advocated for group-based rehabilitation programs, as they provide peer support and camaraderie within these programs [7]. It is important to explore alternative home-based cardiac rehabilitation programs such as telerehabilitation, which can be integrated alongside current models of service delivery and meet an individual’s needs including fostering peer support in a group environment.

## Cardiac telerehabilitation

With advances in new technologies and the exponential growth of the Internet, there are emergent opportunities to deliver cardiac rehabilitation into the home via telerehabilitation. Telerehabilitation is defined as the delivery of rehabilitation services at a distance via telecommunication technology such as phone, videoconferencing and the Internet [16]. Cardiac rehabilitation may be delivered as in-person synchronous, remote, virtual or hybrid programs. As shown in Table 1, these delivery modes are well described by the Million Hearts Cardiac Rehabilitation Think Tank [17].

**Table 1:** Cardiac rehabilitation delivery models

**Delivery modes**	**Definitions**
1) In-person synchronous	Participants and clinicians are co-located at the same time. Examples include traditional centre-based programs and some home-based programs with in-person home visits
2) Telerehabilitation	
a) Remote	Asynchronous activities without real-time communication between participants and clinicians at the time of an exercise session. Exercise occurs at times other than when clinicians and participants are communicating. Examples include text messaging and secure online portals. For instance, the person undertaking cardiac rehabilitation can communicate logged data, such as exercise and/or vital signs, to clinicians over the phone, secure online platforms or via wearables. This may involve physical activity monitoring tools like pedometers, smartphones and implantable cardiac devices accelerometers, and wearable sensors
b) Virtual	Synchronous real-time audio-visual communication between participants and clinicians during an exercise session, but each is in different locations. An example includes videoconferencing
3) Hybrid	A combination of the above models. An example includes an initial centre-based (in-person synchronous) cardiac rehabilitation and then a transition to longer-term maintenance through home-based (virtual) cardiac rehabilitation via videoconferencing

Based on the terminologies recommended by the Million Hearts Cardiac Rehabilitation Think Tank [17]

### Effectiveness of telerehabilitation

There has been a recent proliferation of literature investigating the effects of cardiac telerehabilitation. Chien et al. extend the evidence for remote cardiac rehabilitation in people with HF [18]. In this study, participants in the intervention group were encouraged to undertake home-based strengthening exercises combined with walking for at least 30 min per session, three sessions per week, over 8 weeks. Participants received phone follow-ups every 1 to 2 weeks to monitor progress, provide feedback and solve problems. These authors demonstrated improved quality of life and functional exercise capacity in the intervention group, compared with a control group of maintaining usual activities [18]. Similarly, a 12-week virtual cardiac rehabilitation program delivered by videoconferencing has also been shown to be non-inferior to a traditional centre-based program in terms of functional exercise capacity but has higher attendance rates in individuals with HF [19]. These results indicate that videoconferencing has the benefit of providing direct supervision of a group-based exercise program and enabling real-time audiovisual feedback. Importantly, outcomes from both studies were achieved through readily available off-the-shelf equipment (such as telephone, laptop computer, videoconferencing software, automatic sphygmomanometer and pulse oximeter), which boosts their potential for implementation into clinical settings. One of the key advantages of these cardiac telerehabilitation programs is the reduced transportation. More specifically, participants liked the program convenience, as there was no parking cost and travel time, and thereby lowered the family burden [20]. There are also favourable short-term outcomes with hybrid cardiac rehabilitation in people with HF. The TELEREH-HF study demonstrated improved exercise capacity and quality of life after a 9-week hybrid cardiac rehabilitation program; however, this did not change hospitalisation and mortality rates on long-term follow-up after intervention cessation [21]. A recent systematic review has compared the relative effectiveness of centre-based, home-based, technology-enabled (remote or virtual) and hybrid cardiac rehabilitation in individuals with HF [22]. Of 139 randomised controlled trials with 18,670 participants, cardiac rehabilitation improved functional exercise capacity and quality of life, regardless of the delivery model used [22]. Specifically, this review demonstrated improvements in peak oxygen uptake following centre-based, home-based and technology-enabled programs, with a mean difference (95% credible intervals) of 3.10 (2.56 to 3.65) mL/kg/min, 2.69 (1.67 to 3.70) mL/kg/min and 1.76 (0.27 to 3.26) mL/kg/min respectively, with no significant differences between delivery models [22]. Similarly, there were improvements in the quality of life following centre-based and home-based programs, with a mean difference (95% credible intervals) of − 10.38 (− 14.15 to − 6.46) and − 8.80 (− 13.62 to − 4.07) points respectively on the Minnesota Living with Heart Failure Questionnaire, and no significant differences between delivery models [22]. The authors have also advocated for the selection of delivery models based on the person’s attending preferences, goals and risk stratification [22]. Other systematic reviews have confirmed the cost-effectiveness of telerehabilitation in people with coronary artery disease and HF [23], with incremental cost-effectiveness ratios, ranging from $2099 to $46,972 [24]. Systematic reviews investigating remote, virtual or hybrid cardiac rehabilitation generally report favourable safety data [25, 26].

## Practical considerations on telerehabilitation

From our clinical and research experience, we suggest some practical tips for implementing cardiac telerehabilitation (see Table 2). To maximise the success of telerehabilitation, core components of cardiac rehabilitation should be preserved and aligned with international standards [27–30]. One of the core components is patient assessment including exercise capacity testing [31]. Ideally, this assessment should be undertaken in healthcare facilities for high-risk participants [32]. However, remote assessments have also been recommended for low-risk participants [32]. Some approaches used during the pandemic include estimating exercise capacity with questionnaires, wearables and smart device applications, and directly supervising exercise capacity tests via videoconferencing [33].

**Table 2:** Practical considerations on telerehabilitation implementation

**Key considerations**	**Examples**
Preserve core components of cardiac rehabilitation	Include patient assessment such as exercise capacity testing
Plan	Consider participant eligibility criteria, safety, peer support and monitoring requirements
Prescribe exercise	Apply the same exercise training principles as traditional centre-based programs
Incorporate education	Provide asynchronous education (e.g. access videos and resources via secure e-mail or text messaging) and synchronous education (e.g. real-time interactions during videoconferencing sessions)
Address digital divide	Upskill participants in digital literacy, co-design services and promote wider access to digital technology

One of the commonly reported functional exercise capacity tests used in the home or remote setting is the 6-min walk test. Given the space constraints within the home environment, clinicians may be tempted to undertake a 6-min walk test on a shorter track than the recommended guidelines. However, in a study of individuals with chronic obstructive pulmonary disease, shorter waking distances were recorded with a 10-m track compared with the standard 30-m track [34]. These authors warned that the findings acquired from a shorter track should be interpreted with caution, as studies on prognosis and normative values were generated through tests on longer tracks [34]. Moreover, exercise intensity prescribed based on the 6-min walk test speed may tend to be underestimated when the walk distance is generated on a shorter track.

**Fig. 1:**
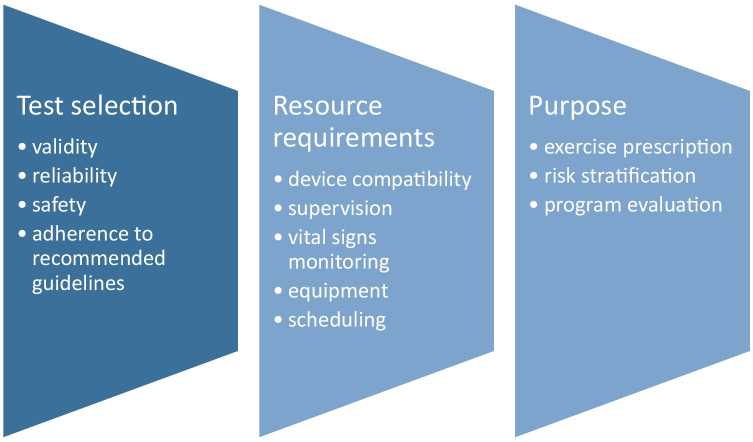
Considerations for undertaking functional exercise capacity tests at home or remotely

As illustrated in Fig. 1, there are other considerations for undertaking exercise capacity tests within the home or remotely. Firstly, choose a test that has demonstrated validity and reliability in the home setting. Secondly, consider device compatibility. Some exercise tests use smart device applications that are unavailable outside of the clinical trial or on particular operating systems. Thirdly, there is a need to balance supervision and resource requirements. For instance, a supervised administration of the test may enable real-time patient communication and safety, but this may have an impact on scheduling, logistics and costs. Lastly, select an exercise test to meet the intended purpose such as guiding exercise prescription, risk stratification and outcome measurement.

In addition to incorporating the core components of cardiac rehabilitation, it is also important to consider the eligibility criteria, safety, peer support and monitoring requirements for cardiac telerehabilitation. Fortunately, Keteyian et al. provide thoughtful insights into these areas [9]. For instance, at the start of each virtual cardiac rehabilitation session, clinicians should confirm the participant’s contact details and location in case emergency services are needed. Strategies to further promote safety include baseline exercise capacity assessment, the presence of a support person and remote monitoring [35]. As it can be challenging to foster peer support in remote cardiac rehabilitation, consider facilitating linkages via support networks and social media. Monitoring in remote cardiac rehabilitation can occur with the participant manually logging the data onto an electronic platform or wearable devices automatically transmitting the data. For virtual cardiac rehabilitation, real-time audio-visual communication and vital signs monitoring can occur via videoconferencing. These non-invasive telemonitoring techniques have been highlighted to reduce morbidity and mortality in people with HF in a recent review [36].

One of the commonly reported barriers to accessing telerehabilitation is digital literacy [37]. It is important to upskill the participant in digital literacy where required. This may involve in-person onboarding, assistance with application downloads and testing operation of wearable devices before the telerehabilitation session. Other strategies proposed to reduce the digital divide include increasing the participant’s knowledge by developing marketing and communication resources in multiple languages and increasing access to technological devices through loan schemes [38].

Another core component of cardiac rehabilitation is exercise training [31]. The same exercise training principles can be applied, regardless of the delivery models. For instance, aim for combined aerobic and strength training as in centre-based cardiac rehabilitation programs, especially for frail participants [32]. Reassuringly, Keteyian et al. have shown that exercise intensity can be performed as prescribed, with no significant difference found between the hybrid (video-based) and the centre-based cardiac rehabilitation groups in their study, with the percentage of participants who trained within their prescribed target heart rate range reported to be 91% and 90% respectively [39].

Education underpins the core components of cardiac rehabilitation including risk factor management and nutritional counselling [28, 29]. Scherrenberg et al. illustrate various options for providing education packages, including asynchronous education where individuals can access videos and resources via secure e-mail or text messaging and synchronous education with a sharing of PowerPoint presentations during videoconferencing sessions [35]. The teach-back method has been recommended in health education to check retention and an individual’s understanding of health information [14]. It comprises of the following components: the healthcare provider delivering health information to the individual; the individual restating the information in their own words; if gaps are identified in the individual’s recap, the cycle of healthcare provider teaching, individual’s restatement and healthcare provider assessment is repeated until the individual accurately comprehends the health information [40]. There is increasing evidence on the use of the teach-back method in health education, with a recent meta-analysis reporting a 40% reduction in overall readmission rates among people with HF [40].

## Pandemic experience and participant preference

During the recent COVID-19 pandemic, cardiac rehabilitation programs have continued to adapt their services through unprecedented levels of innovation and resilience, despite repeated lockdowns and significant staff redeployments. For instance, the UK’s audit data has revealed a significant shift away from the traditional centre-based (72 to 16%) towards home-based cardiac rehabilitation programs (16 to 76%), in the 12-month pre-pandemic to a comparable period during the pandemic [41]. This shift towards technology-enabled (remote or virtual) cardiac rehabilitation programs is echoed by an international survey of 330 clinicians [33]. According to the survey, the telephone remained the most commonly used technology to facilitate the exercise component of cardiac rehabilitation whilst maintaining social distancing and reducing viral transmission, followed by pre-recorded online video, e-mail and videoconferencing [33].

A critical factor in the sustainability of alternative cardiac rehabilitation programs beyond the pandemic is a better understanding of an individual’s preferences. A recent Belgian study has shed some light on an individual’s willingness to participate in these alternative programs [37]. For example, a majority (60%) of participants in cardiac rehabilitation would participate in remote cardiac rehabilitation with an even larger proportion (70%) being interested in hybrid cardiac rehabilitation [37]. An equal proportion of these would prefer centre-based cardiac rehabilitation (44%) compared with either remote or hybrid cardiac rehabilitation (44%). Interestingly, the study showed that only 33% of non-participants in cardiac rehabilitation would be prepared to participate in remote cardiac rehabilitation, and an even smaller proportion (10%) would be prepared to participate in hybrid cardiac rehabilitation [37]. It appears that those already enrolled in cardiac rehabilitation are more prepared to trial alternative models and may reflect their status as having prepared to change their health behaviours [42, 43]. In both participants and non-participants of cardiac rehabilitation, the main facilitator for remote cardiac rehabilitation was the alleviation of transport, and the main barrier was digital literacy. This view is shared by a recent qualitative systematic review on alternative cardiac rehabilitation, which confirmed peer and family support and convenience as facilitators and weather and digital literacy as barriers [44]. To overcome this digital divide, it is therefore important to upskill those wishing to attend remotely in digital literacy, co-design culturally competent services and promote wider access to digital technology [37, 38]. Other suggested strategies to overcome participation barriers include engaging other participants to share stories, seeking technological assistance from family and friends [9] and creating an inclement weather plan [44].

Whilst healthcare professionals are optimistic about retaining hybrid cardiac rehabilitation programs beyond the pandemic, there are opportunities to refine rushed implementation approaches [33, 45]. In a qualitative study on cardiac rehabilitation programs during the pandemic, Australian clinicians identified several challenges with telerehabilitation, including inadequate funding and rapid acquisition of new telehealth equipment and training [45]. This study also highlighted a lack of dedicated space to conduct telerehabilitation as many gym spaces were repurposed for other uses during the pandemic, and capacity challenges with delivering both centre-based and telerehabilitation programs with existing staffing levels [45]. These authors have recommended focusing on the adopter system including workforce training and adequate resources, the organisation including changes in team interactions and routines and the wider context including appropriate funding to ensure the long-term sustainability of these programs [45].

## Directions for future research

Given the expansion of these alternative models of cardiac rehabilitation, a need exists to further build our understanding of the facilitators and barriers of cardiac rehabilitation. Specifically, future research should identify the most effective strategies for promoting program uptake, adherence and completion within different models of care. We also recognise that not all of the programs included in this review meet the full definition of cardiac rehabilitation as established by multiple international organisations. The impact of exercise-only cardiac telerehabilitation programs on morbidity, mortality and readmission is currently unknown and represents a direction for future research. Other identified priority research areas include under-represented populations, patient-centred outcomes, effects on long-term outcomes and cost and implementation in diverse settings [17]. A better understanding of these areas may help to inform future planning for cardiac rehabilitation moving into the post-pandemic era.

## Summary

There has been a growing interest in home-based cardiac rehabilitation programs to widen access and improve program attendance. With rapidly evolving technology and the ubiquitous Internet, there are emergent opportunities to deliver cardiac rehabilitation services into the home via telerehabilitation. Telerehabilitation may be delivered as remote, virtual and hybrid cardiac rehabilitation programs. There is increasing evidence to support cardiac telerehabilitation, with studies generally demonstrating non-inferiority, safety and cost-effectiveness. However, further research should explore the effects of patient-centred and long-term outcomes in diverse populations and settings. With the experience gained during the COVID-19 pandemic, many health services today are in a better position to incorporate telerehabilitation into their usual care and enable greater participant choice.

## Data Availability

Not applicable.
